# Have a novel guideline of American Heart Association outlived usefulness in early differentiated diagnosis of acute aortic dissection

**DOI:** 10.1186/1749-8090-8-S1-P10

**Published:** 2013-09-11

**Authors:** C Park, J Lee, Y Jun, C Choi, K Son, K Park, K Kim, H Cho

**Affiliations:** 1 Department of Thoracic and Cardiovascular Surgery, Gachon University, Gil Hospital, Incheon, Republic of Korea

## Background

Acute aortic dissection (AAD) is a potentially fatal condition that requires rapid assessment and treatment. The American Heart Association and American College of Cardiology suggested new guidelines for early diagnosis of AAD in 2010, and so we applied retrospectively that system to our known patients with AAD in a community hospital.

## Methods

We reviewed 166 patients with confirmed AAD regardless of types from January 2000 to April 2013. We evaluated 12 newly proposed risks, based on the new guideline.

## Results

Abrupt onset of pain was the most frequent symptom (67.4%). 6 patients (3.6%) were grouped under the low risk, 88 patients (53.0%) under the intermediate risk, and 72 patients (43.4%) under the high risk. 90 patients (54.2%) demonstrated a widened mediastinum in the chest X-rays. 3 patients showed a mediastinal widening among 6 patients with low risk. In 7 patients (4.2%), were initially diagnosed with acute myocardial infarction, 3 patients were categorized as intermediate risk group (risk score 1) and the others as a high risk of AAD (risk score 2).

## Conclusions

The risk score system in new guidelines detected ADD with high sensitivity. But, in addition to applying the new guideline, having high suspicion for AAD enables the early diagnosis to be more accurate.

